# EphA2 promotes tumorigenicity of cervical cancer by up‐regulating CDK6

**DOI:** 10.1111/jcmm.16337

**Published:** 2021-02-14

**Authors:** Changhao Huang, Zihua Chen, Yihong He, Zhengxi He, Zhenying Ban, Yuanhang Zhu, Leilei Ding, Chen Yang, Ji‐Hak Jeong, Weijie Yuan, Li Yang

**Affiliations:** ^1^ Hunan Key Laboratory of Precise Diagnosis and Treatment of Gastrointestinal Tumor Xiangya Hospital Central South University Changsha China; ^2^ Department of Gastrointestinal Surgery Xiangya Hospital Central South University Changsha China; ^3^ Department of Oncology Xiangya Hospital Central South University Changsha China; ^4^ The Third Affiliated Hospital of Zhengzhou University Zhengzhou China; ^5^ Research Institute of Pharmaceutical Sciences Kyungpook National University Daegu Korea

**Keywords:** CDK6, cervical cancer, chemotherapy resistance, EphA2

## Abstract

Erythropoietin‐producing hepatocellular receptor A2 (EphA2) receptor tyrosine kinase plays an important role in tissue organization and homeostasis in normal organs. EphA2 is overexpressed in a variety of types of solid tumours with oncogenic functions. However, the role of EphA2 in cervical cancer (CC) is still needed to be further explored. Here, we examined the role of EphA2 by establishing a stable EphA2 knock‐down CC cell lines or a stable EphA2‐overexpressed CC cells lines. Overexpression of EphA2 increased cell proliferation and migration of CC while EphA2 knock‐down decreased the CC tumorigenicity. In addition, EphA2 knock‐down suppressed CC tumour development in the xenograft mouse model. Inhibition of EphA2 by AWL‐II‐41‐27, EphA2‐specific tyrosine kinase inhibitor, or knock‐down of EphA2 decreased mRNA and protein expression of cyclin‐dependent kinase (CDK) 6 in CC cells, which increased cellular susceptibility to epirubicin (EPI), an anti‐cancer chemotherapy drug. A clinicopathological study of EphA2 was conducted on a cohort of 158 human CC patients. EphA2 protein expression was positively correlated with CDK6 protein expression, invasion depth, lymph node metastasis and clinicopathological stage (*P* < .05). This study demonstrates the oncogenic activity of EphA2 in vitro and in vivo, which provides insights into the relevant mechanisms that might lead to novel treatments for CC.

## 1 INTRODUCTION

Cervical cancer (CC) is a major gynaecologic cancer worldwide and ranks second mortality in women. CC accounts for a large proportion of cancer‐associated mortality.^1^ The decline in CC incidence is largely due to the widespread use of cytology screening; however, invasive CC still remains a considerable threat to the lives of women. Although CC patients in their early stages could often benefit from standard treatments of chemotherapy and radiotherapy, which significantly improve clinical outcome, the development of clinical management still remains a challenge, especially in advanced‐stage CC.^2^ Epirubicin (EPI) is an initial chemotherapy drug for advanced and metastasis CC patients.^3^ Over 50% of advanced CC patients eventually relapse despite their initial response to chemotherapy.^4^ Therefore, the chemotherapy resistance is the key barrier to improving therapy efficacy in CC patients.

Erythropoietin‐producing hepatocellular A2 (EphA2) is a key member of Eph receptors, which is one of the largest subfamily of receptor tyrosine kinases (RTKs).^5^ EphA2 is frequently detected at low levels in various normal epithelial cells, and its overexpression has been investigated in solid tumours, including glioma,^6^ non‐small cell lung cancer,^7^ gastric cancer,^8^ renal cell cancer,^9^ colorectal cancer and endometrial cancer.^10^ Previously, we reported that the overexpression of EphA2 was related to poor prognosis of patients with gastric cancer and promotes proliferation through Wnt/β‐catenin and Hippo pathways of gastric cancer cells.^8^, ^11^ However, the exact mechanism is still unclear by which EphA2 plays a role in the development of CC and treatment of drug resistance in CC.

Cyclin‐dependent kinases (CDKs) are a family of protein kinases involved in cell division, apoptosis and neurogenesis.^12^ CDK6 is a member of the CDK family, which responsible cell cycle of G1 to S regulation and cell differentiation.^13^ CDK6 has been reported in many solid tumours, such as colorectal carcinoma, medulloblastoma and oral squamous cell carcinomas.^12^, ^14^ After the inhibitors of CDK6 were introduced into clinic treatment strategy in advanced breast cancer, the patients got more benefits from chemotherapy.^15^ Mitogenic forces, like oestrogen receptor transcriptional activity and tyrosine kinase signalling including ERBB2, phosphoinositide 3‐kinase/AKT and mammalian target of rapamycin, can make cyclin D1 levels be increased, thereby CDK4/6 activated, promoted cellular progression to S phase.^16^ However, it is not reported that the Eph family members regulate CDK6 expression in CC.

In this study, we examined that EphA2 promoted tumorigenicity of CC in vivo and in vitro. EphA2 increased cell proliferation and migration and led to chemoresistance of CC to EPI, which was mediated with the up‐regulation of CDK6. In addition, we demonstrated the correlation between the protein expression levels of EphA2 and CDK6 in CC patients by immunohistochemical staining of tissue specimens. This study will provide a novel therapeutic strategy for CC treatment.

## 2 METHOD AND MATERIAL

### 2.1 Cells and reagents

SiHa, HeLa and C4‐i cells were cultured in 1640 (C4‐i and HeLa) and DMEM (SiHa) supplemented with 10% foetal bovine serum (FBS) and 1% antibiotic‐antimycotic and incubated at 37°C in a moist atmosphere containing 5% CO_2_. ALW‐II‐41‐27 (ALW) was purchased from MedChemExpress. Epirubicin (EPI) was purchased from Sigma‐Aldrich.

### 2.2 Transduction and transfection

When SiHa cells reached 75%‐90% confluence on the day of transduction, two lentiviral stocks (EphA2‐shRNA and control shRNA, purchased from Sigma) were transduced into the cells with polyethylenimine (PEI). Twenty‐four hours after infecting, the cells were dispersed into a 10 cm culture dish with antibiotic (puromycin). When C4‐i or HeLa cells reached 80%‐90% confluence, PEI was used to transfect plasmids (EphA2‐overexpressing plasmid and control empty vector plasmids). Twenty‐four hours after infecting, the cells were dispersed into a 10 cm culture dish with antibiotic (puromycin) (hygromycin). After 2‐3 weeks later, the cell colonies were collected, and Western blot analysis was collected to analyse the level of EphA2 expression.

### 2.3 Western blot analysis

Anti‐EphA2 antibody (#6997) and anti‐CDK6 antibody (#13331) were purchased from CST. The transferred membranes blocked with 5% BSA were incubated more than 16 hours at 4°C with the primary (1:1000) and secondary antibodies (1:3000) for 1 hour in sequence. Pearce ECL Western blotting Substrate (purchased from Thermo Scientific) was explored for chemiluminescence detection.

### 2.4 Cell proliferation assay

The cervical cancer cells were inoculated in 96‐well plates with 4 × 10^3^ cells per well and cultured 8‐12 hours. At different time‐points, 10 µL 3‐(4,5‐dimethylthiazol‐2‐yl)‐2,5‐diphenyltetrazolium bromide (MTT) dye was added, incubated for 3.5 hours at 37°C, and the original medium was removed. One hundred microliter DMSO shaker 10 minutes was added to each well. A microboard reader (Tecan) was used to measure the spectrometric absorbance at the wavelengths of 570 and 630 nm.

### 2.5 Wound‐healing assay

The cells at 95% laying fusion were wounded by dragging a line with a 10 µL pipette tip. The cells were washed by PBS for three times to remove cellular debris, incubated for 12‐24 hours, and the images were captured using an inverted microscope.

### 2.6 Tumour sphere formation assay

The cells (1 × 10^2^ cells/mL) were inoculated into each well of a 24‐well ultra‐low attachment plate and treated in a serum‐free DMEM/F12 medium (Invitrogen) supplemented with 15‐20 ng/mL basic fibroblast growth factor, 15‐20 ng/mL epidermal growth factor (R&D Systems), 3‐5 µg/mL insulin (Sigma) and B‐27 Supplement (Life technologies), and about 20% of the medium was changed every 3‐5 days. The images were obtained using an inverted bright‐field microscope.

### 2.7 Transwell migration assay

Transwell invasion assays were carried out on a 24‐well 8 µm‐pore‐size Transwell plates coated with Matrigel Basement Membrane Matrix (BD Bioscience) on the bottom. Cells (5 × 10^4^) were added in the upper side of the insert treating with medium containing 1% FBS. In the lower chamber, the medium containing 10%‐15% FBS was used as chemoattractant.

### 2.8 Quantitative real‐time PCR(q‐rtPCR)

Synthesis of cDNA by extracting 1 μg total RNA with TRIzol (Invitrogen) and TaqMan Reverse Transcription Reagent. The relative expression of target gene mRNA was expressed by the ratio of target β‐actin. The sequences for the primers of CDK6 are 5′‐GCTGACCAGCAGTACGAATG‐3′ and 5′‐GCACACATCAAACAACCTGACC‐3′. The relative expression amount of target gene mRNA was calculated directly according to the standard curve.

### 2.9 Animal studies

Four‐week‐old female NOD SCID mice were injected in their backside subcutaneously with SiHa‐EphA2‐KD, SiHa‐Con, C4‐i‐Eph2‐OE or C4‐i‐Con cells (1 × 10^6^ cells in 200 µL culture media), and the tumour development was monitored. After 3 weeks for SiHa cells or 4 weeks for C4‐I cells, the mice were sacrificed, and the tumour was collected, and the tumour size and weight were measured.

### 2.10 Patient selection and pathologic tissue preparation

Between December 2017 and December 2018, 158 patients with CC, 103 patients with cervical intraepithelial neoplasia (CIN) and 58 patients with cervicitis admitted to the 3rd Affiliated Hospital of Zhengzhou University (Zhengzhou, China) were selected. The study got permission from the Research Ethics Committee of this hospital. The various tissues were removed, fixed with 10% formalin and embedded in 65℃ paraffin blocks. Each sample was prepared with thickness of 4 µm, dewaxing and rehydration.

### 2.11 Immunohistochemistry

Paraffin‐embedded CC tissue sections were stained with EphA2 (Santa Cruz sc‐398832, 1:200) and CDK6 (Santa Cruz sc‐7961 1:400) immunohistochemical staining. Non‐immune goat serum was used instead of primary antibody to prepare negative control. Two independent pathologists, respectively, evaluated the staining results. Means were taken for final analysis. The samples in which the staining intensity was none or weak and less than half cells were stained were rendered negative (−), while the samples with moderate or strong staining in more than half cells were positive.

### 2.12 Availability of data

The data will be made available upon reasonable request.

### 2.13 Statistical analysis

Differences between groups were examined for statistical significance using Student's *t* test. All *P* values are two tailed, and *P* values <.05 were considered to indicate statistical significance.

## 3 RESULT

### 3.1 The knock‐down of EphA2 decreased CC tumorigenicity in vitro and vivo

**Figure 1:**
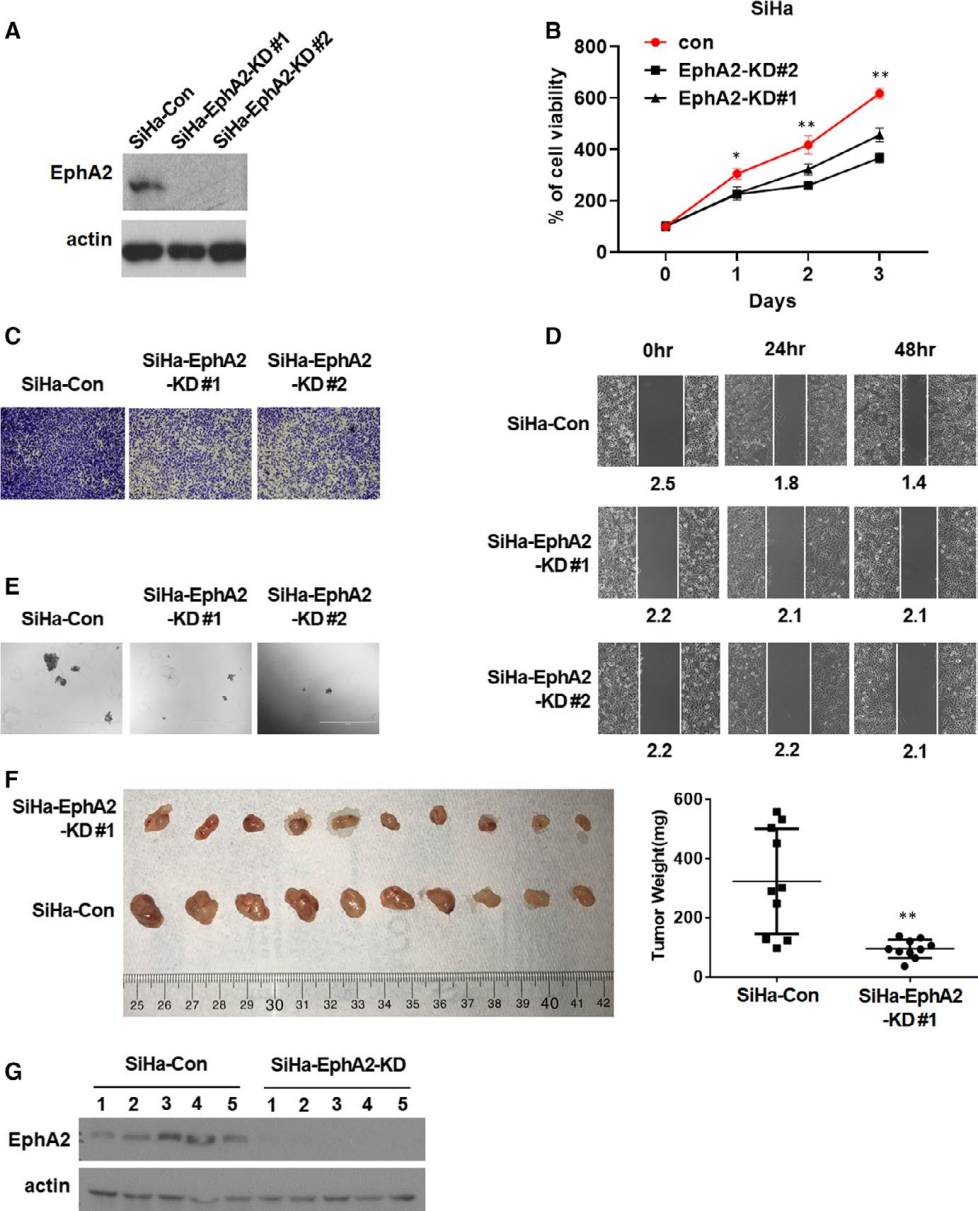
The knock‐down of EphA2 decreased tumorigenic cell growth of CC and inhibited CC xenograft tumour growth. A, Western blot analysis of EphA2 protein expression in SiHa cells transfected with EphA2 shRNA (SiHa‐EphA2‐KD#1, SiHa‐EphA2‐KD#2) and control shRNA (SiHa‐Con). B, MTT analysis for cell proliferation of SiHa cells transfected with EphA2 shRNA (SiHa‐EphA2‐KD#1, SiHa‐EphA2‐KD#2) and control shRNA (SiHa‐Con). C, Transwell migration assay of SiHa cells transfected with EphA2 shRNA (SiHa‐EphA2‐KD#1, SiHa‐EphA2‐KD#2) and control shRNA (SiHa‐Con). D, Wound‐healing assay of SiHa cells transfected with EphA2 shRNA (SiHa‐EphA2‐KD#1, SiHa‐EphA2‐KD#2) and control shRNA (SiHa‐Con). E, Tumour sphere assay of SiHa cells transfected with EphA2 shRNA (SiHa‐EphA2‐KD#1, SiHa‐EphA2‐KD#2) and control shRNA (SiHa‐Con). F, Xenograft tumour development in NOD SCID mice inoculated with SiHa cells transfected with EphA2 shRNA (SiHa‐EphA2‐KD#1) or control shRNA (SiHa‐Con) (n = 10). Four weeks later, mice were sacrificed, and tumours were collected (left) and weighted. G, Western blot analysis of EphA2 protein expression in the tumours from Figure 1F. The results in (B) and (F) represent the mean ± SD. Statistically significant differences are indicated. **P < .01

To examine the role of EphA2 in CC, we first established EphA2 knock‐down cell lines (SiHa‐EphA2‐KD, SiHa‐Con). Knock‐down efficiency of EphA2 protein in these cells was determined by Western blot analysis (Figure 1A). We next examined the in vitro tumorigenicity of EphA2 using MTT assay, transwell migration assay, wound‐healing assay and tumour sphere formation assay. We found that the knock‐down of EphA2 significantly reduced cell proliferation (Figure 1B), transwell migration (Figure 1C and Figure S1A), cell migration (Figure 1D) and tumour sphere formation (Figure 1E and Figure S1B). To further test the tumorigenic activity of EphA2 in vivo, we employed a mouse xenograft model. NOD SCID mice were inoculated with SiHa‐EphA2‐KD and SiHa‐Con cells and monitored the tumour development. The mice were sacrificed after 3 weeks, and the tumours were collected and weighted (Figure 1F). We found that the knock‐down of EphA2 significantly decreased CC tumour development (Figure 1F). Knock‐down efficiency of EphA2 protein in the tumours was determined by Western blot analysis (Figure 1G). These results indicate that knock‐down of EphA2 suppressed CC tumorigenicity in vitro and vivo.

### 3.2 The overexpression of EphA2 promotes CC tumorigenicity in vitro and in vivo

**Figure 2:**
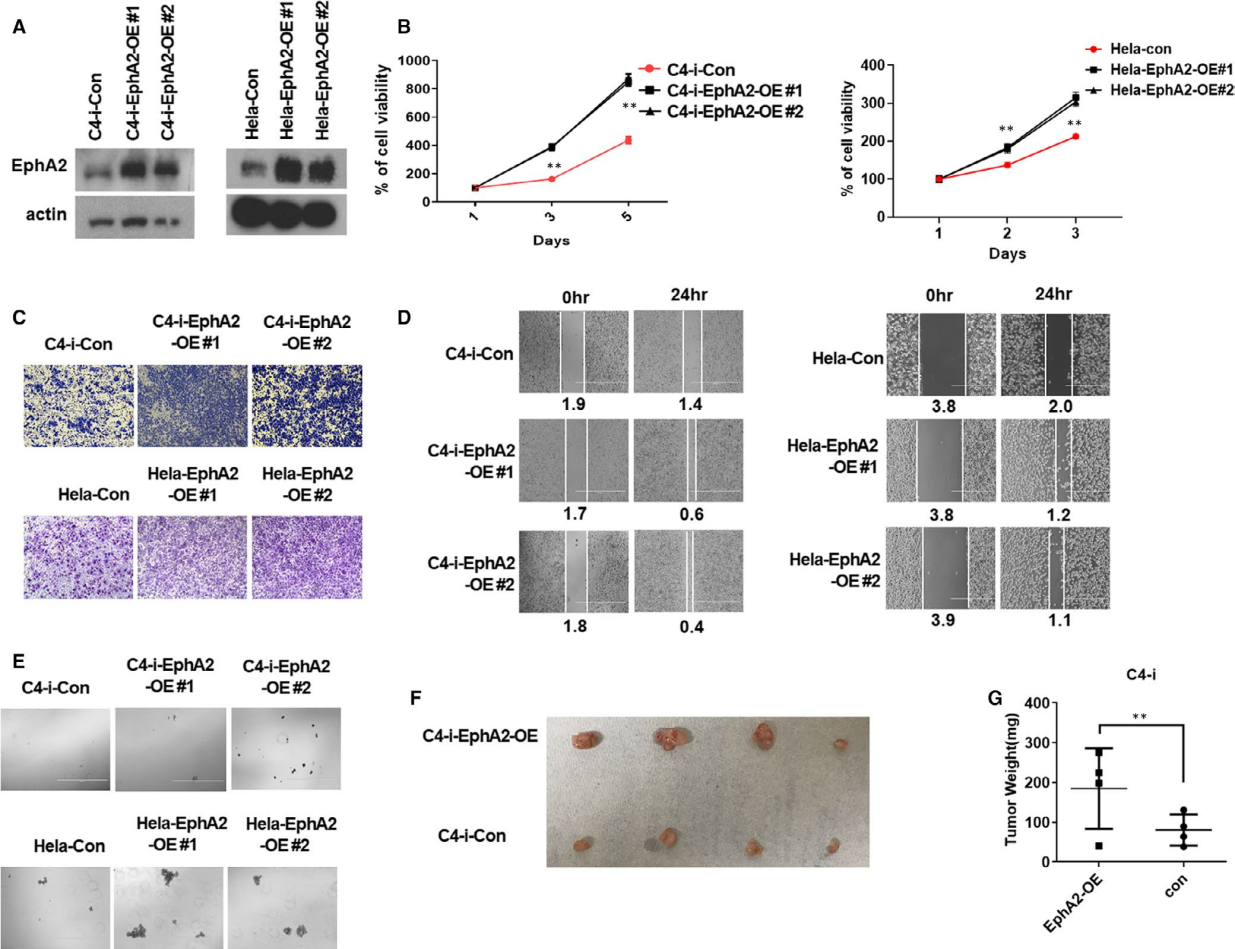
The overexpression of EphA2 increased tumorigenic cell growth of CC. A, Western blot analysis of EphA2 protein expression in C4‐i and HeLa cells transfected with EphA2 expression plasmid (C4‐i‐EphA2‐OE#1, C4‐i‐EphA2‐OE#2, HeLa‐EphA2‐OE#1, HeLa‐EphA2‐OE#2) and control vector (C4‐i‐Con, HeLa‐Con). B, MTT analysis for cell proliferation of C4‐i cells transfected with EphA2 expression plasmid (C4‐i‐EphA2‐OE#1, C4‐i‐EphA2‐OE#2, HeLa‐EphA2‐OE#1, HeLa‐EphA2‐OE#2) and control vector (C4‐i‐Con, HeLa‐Con). C, Transwell migration assay of C4‐i or Hela cells transfected with EphA2 expression plasmid (C4‐i‐EphA2‐OE#1, C4‐i‐EphA2‐OE#2, HeLa‐EphA2‐OE#1, HeLa‐EphA2‐OE#2) and control vector (C4‐i‐Con, HeLa‐Con). D, Wound‐healing assay of C4‐i cells transfected with EphA2 expression plasmid (C4‐i‐EphA2‐OE#1, C4‐i‐EphA2‐OE#2, HeLa‐EphA2‐OE#1, HeLa‐EphA2‐OE#2) and control vector (C4‐i‐Con, HeLa‐Con). E, Tumour sphere assay of C4‐i cells transfected with EphA2 expression plasmid (C4‐i‐EphA2‐OE#1, C4‐i‐EphA2‐OE#2, HeLa‐EphA2‐OE#1, HeLa‐EphA2‐OE#2) and control vector (C4‐i‐Con, HeLa‐Con). F, Xenograft tumour development in NOD SCID mice inoculated with control (C4‐i‐Con) or EphA2 overexpression (C4‐i‐EphA2‐OE) cells (n = 4). Four weeks later, mice were sacrificed, and tumours were collected (left) and the volume was calculated (right). G, The tumours weight was calculated from Figure 2F. The results in (B), (F), and (G) represent the mean ± SD. Statistically significant differences are indicated.    **P < .01

To further investigate the role of EphA2 in CC, we established EphA2 overexpression cell lines (C4‐i‐EphA2‐OE, C4‐i‐Con, HeLa‐EphA2‐OE and HeLa‐Con). The level of EphA2 protein expression in these cells was determined by Western blot analysis (Figure 2A). We examined the in vitro tumorigenicity in these cells using the various assays as same as above. We found that the overexpression of EphA2 significantly increased cell proliferation (Figure 2B), colony formation (Figure 2C and Figure S2A), cell migration (Figure 2D) and tumour sphere formation (Figure 2E and Figure S2B). We next examined the in vivo tumorigenicity using xenograft experiment. NOD SCID mice were subcutaneously inoculated with C4‐i‐Con or C4‐i‐Eph2‐OE cells in the flanks, and the tumour development was monitored. We found that EphA2 overexpression significantly increased tumour size (Figure 2F) and weight (Figure 2G) in NOD SCID mice. These results suggest that overexpression of EphA2 increased CC tumorigenicity in vivo and in vitro.

### 3.3 EphA2 conferred CC cell chemotherapy resistance by up‐regulating CDK6 expression

**Figure 3:**
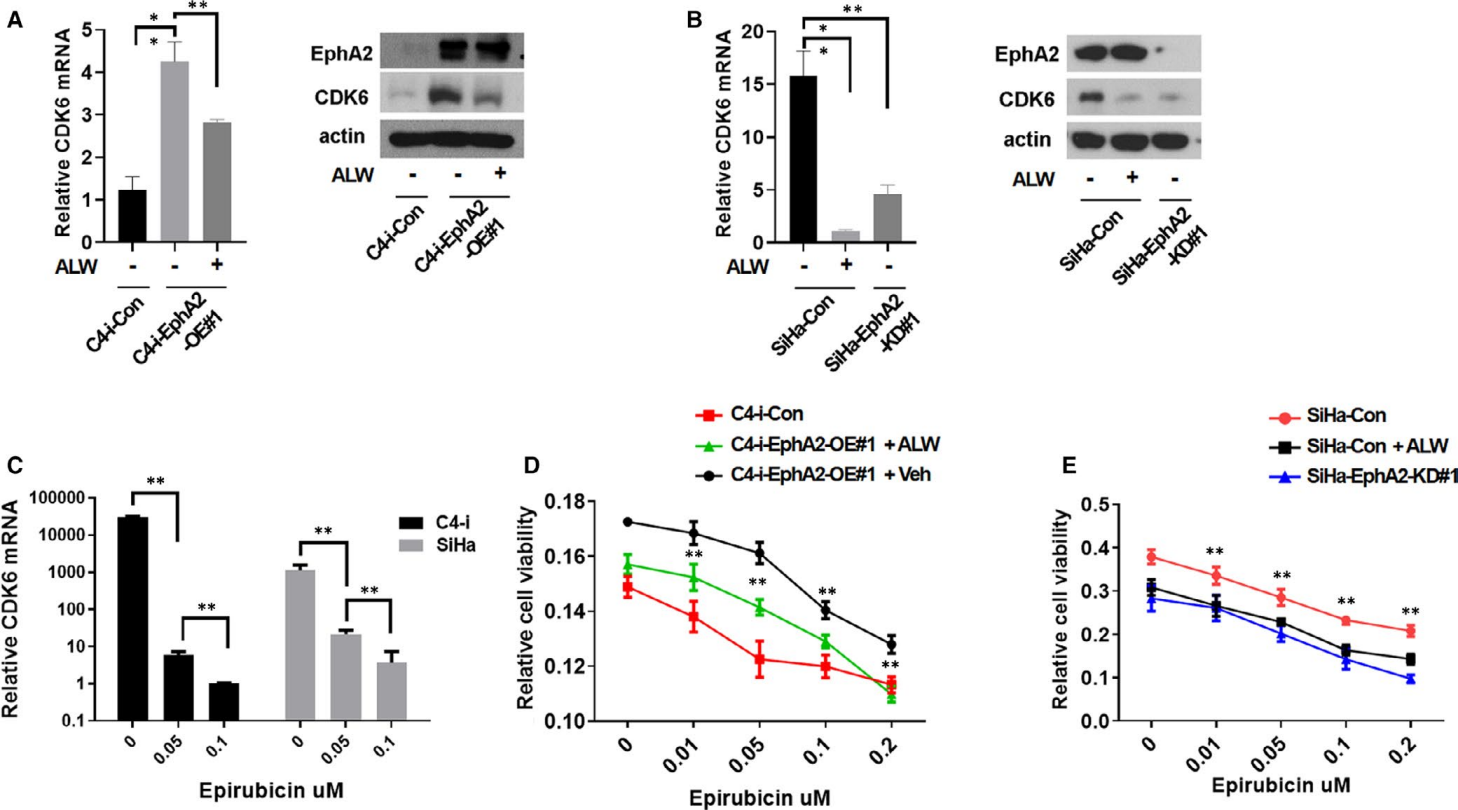
EphA2 regulated CC cell chemotherapy resistance by inducing CDK6 expression. A, Real‐time PCR analysis of CDK6 mRNA expression (left) and Western blot analysis of CDK6 protein expression (right) by treatment of ALW (0.5 µmol/L) for 48 h in C4‐i cells transfected with EphA2 expression plasmid (C4‐i‐EphA2‐OE#1) and control vector (C4‐i‐Con). B, Real‐time PCR analysis of CDK6 mRNA expression (left) and Western blot analysis of CDK6 protein expression (right) by treatment of ALW (0.5 µmol/L) for 48 h in SiHa cells transfected with EphA2 shRNA (SiHa‐EphA2‐KD#1) and control shRNA (SiHa‐Con). C, Real‐time PCR analysis of CDK6 mRNA expression by treatment of ALW at various concentrations for 48 h in C4‐i and SiHa cells. D, MTT analysis for cell proliferation by treatment of ALW (0.5 µmol/L) for 48 h in C4‐i cells transfected with EphA2 expression plasmid (C4‐i‐EphA2‐OE#1) and control vector (C4‐i‐Con). E, MTT analysis for cell proliferation by treatment of ALW (0.5 µmol/L) for 48 h in SiHa cells transfected with EphA2 shRNA (SiHa‐EphA2‐KD#1) and control shRNA (SiHa‐Con). The results in (A‐E) represent the mean ± SD. Statistically significant differences are indicated. *P < .05, **P < .01

CDK6 plays an important role in regulating the cell cycle progression. However, there were no reports for the relation between CDK6 and EphA2. To test the regulation of CDK6 by EphA2 in CC, we employed ALW that is EphA2 inhibitor specifically targeting Ser897 phosphorylation.^17^ We investigated the level of CDK6 mRNA and protein by treatment of ALW in EphA2 overexpression or knock‐down CC cell lines. EphA2 overexpression increased the level of CDK6 mRNA and protein (Figure 3A) while EphA2 knock‐down decreased them (Figure 3B). In addition, the treatment of ALW decreased the levels of CDK6 mRNA and protein in both C4‐i‐EphA2‐OE cells (Figure 3A) and SiHa‐Con cells (Figure 3B). These results suggest that EphA2 regulates the expression of CDK6 at the transcriptional level. At present, for standard chemotherapy regimens for the treatment of CC, one of them is EPI alone or in combination with other conventional drugs, but its efficacy is limited due to the frequent occurrence of drug resistance.^18^ We first examined CDK6 mRNA expression by treatment of EPI in C4‐I or SiHa cell lines. We found that the expression of CDK6 mRNA was decreased by EPI treatment in a dose‐dependent manner in these cell lines (Figure 3C). To test the drug resistance of EphA2 to EPI, we examined cell viability by EPI treatment in EphA2 overexpression or knock‐down CC cell lines. We found that the overexpression of EphA2 could significantly increase the resistance of CC cells to EPI (Figure 3D) whereas the EphA2 knock‐down decreased the resistance of CC cells to EPI (Figure 3E). In addition, we also found that ALW could significantly increase the susceptibility of CC cells to EPI (Figure 3D,E). These results suggest that EphA2 regulates the chemotherapy resistance of CC cells by inducing CDK6 expression.

In addition, we investigated the effects of CDK6 overexpression on the oncogenic activity in EphA2 knock‐down cells. We first overexpressed CDK6 in SiHa‐EphA2‐KD cells and established the EphA2‐KD/CDK6‐OE cells (Figure S3A). We next examined the oncogenic activity including proliferation, migration, sphere formation, and chemotherapy resistance using the EphA2‐KD/CDK6‐OE cells. We found that CDK6 overexpression in EphA2 knock‐down cells significantly restored cell proliferation (Figure S3B), migration (Figure S3C) and sphere formation (Figure S3D), which are similar to the control. In addition, we also found that the resistance to EPI of EphA2‐KD/CDK6‐OE cells was significantly increased compared to that of EphA2‐KD cells (Figure S3E). Collectively, these results suggest that CDK6 overexpression in EphA2 knock‐down cells restores oncogenic activity of the cervical cancer cells.

### 3.4 EphA2 levels were correlated with CDK6 levels in human CC

**Figure 4:**
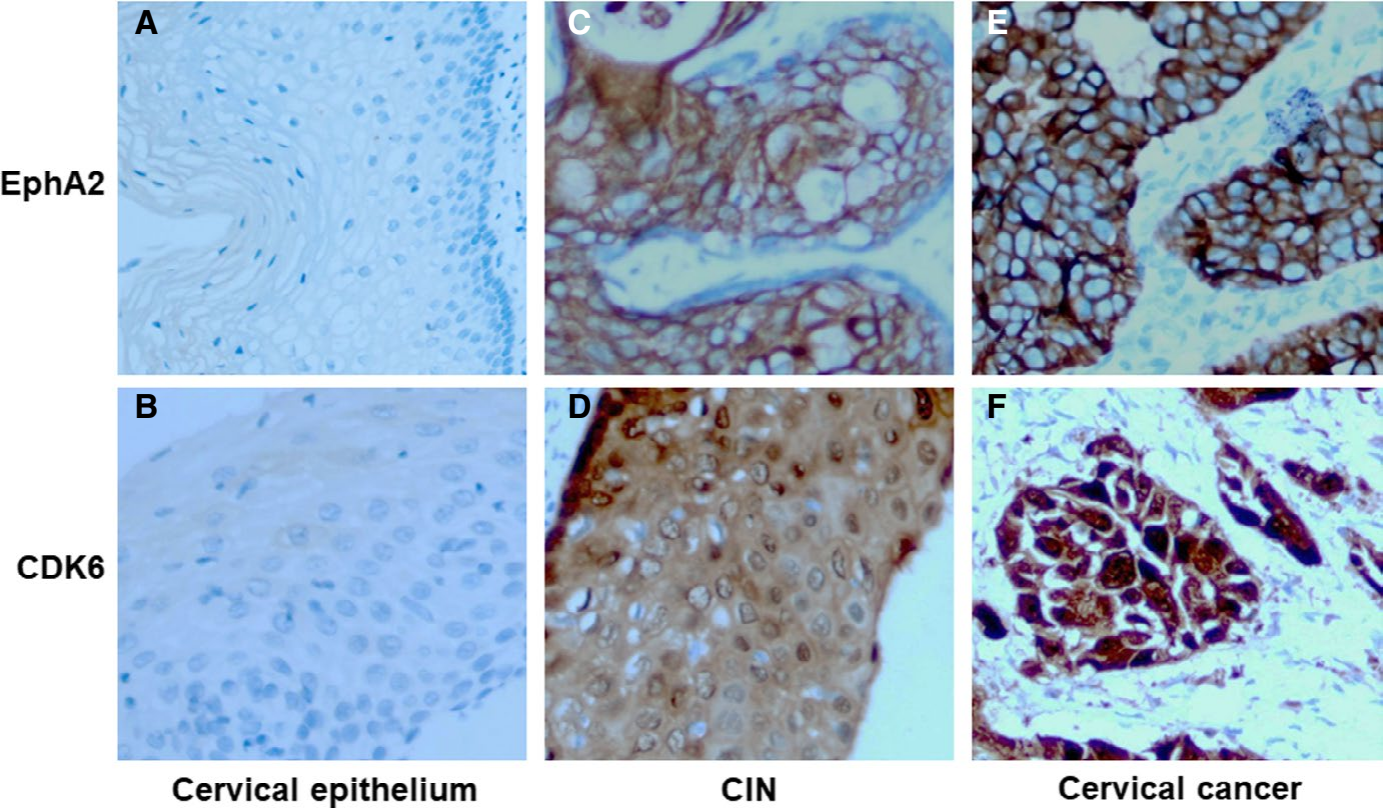
Clinical relevance of EphA2 and CDK6 in CC. Representative staining of immunohistochemical analysis for the protein expression of EphA2 and CDK6 in human normal cervical epithelium (A, B), CIN (C, D) and CC (E, F)

We analysed 158 cases of CC, 103 cases of CIN and 58 cases of normal cervical epithelium by immunohistochemistry to examined the EphA2 and CDK6 proteins expression (Figure 4A‐F). We found that the expression of EphA2 and CDK6 protein was correlated with the clinical characteristics (Table 1). The expression level of EphA2 and CDK6 protein in the CC tissues were significantly higher than that in the cervical epithelium and CIN (Table 1). In addition, there was a significantly correlation between EphA2 protein and CDK6 protein expression in human cervical cancer tissues (Table 2). These results suggest that the correlation between EphA2 protein and CDK6 protein expression is manifested in human cervical cancer.

**Table 1:** The Correlation of the staining of EphA2 and CDK6 in normal cervical epithelial, cervical intraepithelial neoplasia (CIN) and advanced cervical cancer (chi‐square test)

	n	EphA2			CDK6		
		+	−	*P*	+	−	*P*
Age							
<45	165	58	107	<.01	62	103	<.01
≥45	154	93	61		108	46	
Normal cervical epithelial	58	7	51	<.01	9	49	<.01
CIN	103	24	79		31	72	
Cervical cancer	158	120	38		132	26	
Cervical cancer in situ	36	20	16	<.01	27	9	Ns
Advanced cervical cancer	122	100	22		105	17	
Histologic type							
Poor and undifferentiated	72	56	16	Ns	59	13	Ns
Well and moderate	50	44	6		46	4	
Stage							
Ⅰ	36	22	14	<.01	31	5	Ns
Ⅱ	86	78	8		74	12	
Lymph node metastasis							
−	64	47	17	<.05	85	10	Ns
+	58	53	5		20	7	

**Table 2:** Correlation between the staining of EphA2 and CDK6 in Cervical intraepithelial neoplasia (CIN) and advanced cervical cancer tissue (Pearson test)

		CDK6		*r*	*P*
		−	+		
CIN	EphA2				
	−	69	10	.690	<.01
	+	3	21		
Advanced cervical cancer	EphA2				
	−	16	6	.796	<.01
	+	1	99		

## 4 DISCUSSION

RTK regulates a variety of cellular processes and downstream signal transduction pathways. EphA2, as one of the well‐studied RTKs, has been a focus on cancer research for many years.^9^ Increasing evidence indicated that the EphA2 plays an important role in cell transformation, primary tumour progression, angiogenesis and metastasis in various tumour models.^6^, ^7^, ^8^ However, its exact function in CC has not been studied. The high expression of EphA2 and EphrinA‐1 protein in cervical squamous cell carcinoma indicates a poorer overall survival,^19^, ^20^ but the underlying mechanism remains largely unknown. Here we showed that the level of EphA2 expression in CC cell lines was positively correlated with tumour cell proliferation, migration and invasion abilities, which was demonstrated by in vivo and in vitro experiments. EphA2 overexpression could lead to the up‐regulation of CDK6 mRNA and protein expression, which results in promoting the abovementioned oncogenic functions and cell resistance to EPI. In addition, clinical pathological analysis revealed that the overexpression of EphA2 in CC tissue specimen was positively correlated with the tumour stage and lymph node metastasis.

We previously reported that EphA2 is frequently overexpressed and correlated with increased cell proliferation, invasion and migration ability of gastrointestinal tumours.^8^, ^11^ The overexpression of EphA2 in tumours is related to tumour metastasis and poor prognosis of patients, which is caused by epithelial‐mesenchymal transition (EMT) of tumour cells. Tumour cells with EMT show characteristics that promote distal metastasis and chemotherapy resistance, including cell death resistance and senescence, evasion of immune surveillance and acquisition of stem cell properties.^21^ EphA2 could activate Wnt/beta‐catenin and Hippo signalling pathways, which in turn induced EMT and downstream signalling cascade, thus mediating tumour invasiveness and resistance to cancer therapeutics in gastric cancer.^8^, ^11^ Similar to CC, the prognosis of patients becomes significantly poor as the tumour metastases to other parts of the body, in which EMT plays an important role.^22^ EMT causes chemoresistance and radio‐resistance in CC cells. The inhibition of EMT make CC cells be more sensitive to radiation and drugs. This sensitization could improve the survival rate of CC patients.

Interestingly, we found that the expression of EphA2 could lead to the regulation of CDK6 mRNA and protein expression in CC. It has been found that CDK6 plays an important regulatory role in the process of cell cycle.^12^ Previous studies have shown that the up‐regulation of CDK6 activity is related to the occurrence of several cancers.^16^ CDK6 in the G1 phase of cell cycle can phosphorylate the retinoblastoma (Rb) and its related proteins, and relieve the inhibition of E2F. Then, E2F encodes the proteins required for DNA replication (S phase entry) by activating gene transcription. The CDK6 activation requires binding to D‐type cyclins, which is phosphorylated by CDK‐activated kinase (CDK7/cyclin H/MAT1). CDK6 and Cip/Kip proteins could be deactivated by INK4s, as negative modulators of the CDK6‐cyclin D complex. MicroRNAs (MiRNAs) blocking the expression of CDK6, which can inhibit the proliferation of many solid tumour cells, such as gliomas, medulloblastoma, prostate, bladder, gastric, hepatocellular and lung, suggesting that CDK6 plays an important role in the development of these tumours.^16^, ^23^, ^24^, ^25^ The mechanism of treatment by which targets CDK6 through inhibiting miRNA function has been proven in CC.^26^, ^27^, ^28^ We provided evidence that inhibiting EphA2 tyrosine kinase activity by specific tyrosine kinase inhibitor (TKI) could also decrease CDK6 expression, indicating that the combination of both inhibitors might provide potential benefits to CC patients with high EphA2 protein expression.

In summary, we reported that the level of EphA2 expression is positively correlated with CC tumour proliferation, invasion, patient overall survival and therapeutic resistance of CC to EPI. EphA2 could be an therapeutic target for the treatment of CC. Combinational therapy targeting EphA2 with conventional chemotherapy drugs might provide therapeutic benefits to CC patients.

## CONFLICT OF INTEREST

No potential conflicts of interest were disclosed.

## AUTHOR CONTRIBUTIONS

**Changhao Huang:** Methodology (lead); Project administration (lead); Writing‐original draft (lead). **Z. Chen:** Supervision (lead). **Zhengxi He:** Writing‐review & editing (equal). **Yihong He:** Funding acquisition (equal); Writing‐review & editing (supporting). **Leilei Ding:** Data curation (equal). **Yuanhang Zhu:** Formal analysis (equal). **Zhenying Ban:** Validation (equal). **Chen Yang:** Writing‐original draft (equal). **Ji‐hak Jeong:** Writing‐review & editing (equal). **Weijie Yuan:** Supervision (supporting); Visualization (supporting). **Li Yang:** Conceptualization (supporting); Investigation (supporting).

## Supporting information

Supplementary Material

## References

[1] Small W , Bacon MA , Bajaj A , et al. Cervical cancer: a global health crisis. Cancer. 2017;123:2404‐2412. 28464289 10.1002/cncr.30667

[2] Wang Y , Farmer M , Izaguirre EW , et al. Association of definitive pelvic radiation therapy with survival among patients with newly diagnosed metastatic cervical cancer. JAMA Oncol. 2018;4:1288‐1291. 30054609 10.1001/jamaoncol.2018.2677 PMC6143011

[3] Evangelatov A , Skrobanska R , Mladenov N , Petkova M , Yordanov G , Pankov R . Epirubicin loading in poly(butyl cyanoacrylate) nanoparticles manifests via altered intracellular localization and cellular response in cervical carcinoma (HeLa) cells. Drug Deliv. 2016;23:2235‐2244. 25268149 10.3109/10717544.2014.962117

[4] Ventriglia J , Paciolla I , Pisano C , et al. Immunotherapy in ovarian, endometrial and cervical cancer: State of the art and future perspectives. Cancer Treat Rev. 2017;59:109‐116. 28800469 10.1016/j.ctrv.2017.07.008

[5] Pasquale EB . Eph receptors and ephrins in cancer: bidirectional signalling and beyond. Nat Rev Cancer. 2010;10:165‐180. 20179713 10.1038/nrc2806 PMC2921274

[6] Binda E , Visioli A , Giani F , et al. The EphA2 receptor drives self‐renewal and tumorigenicity in stem‐like tumor‐propagating cells from human glioblastomas. Cancer Cell. 2012;22:765‐780. 23238013 10.1016/j.ccr.2012.11.005 PMC3922047

[7] Zhou Y , Yamada N , Tanaka T , et al. Crucial roles of RSK in cell motility by catalysing serine phosphorylation of EphA2. Nat Commun. 2015;6:7679. 26158630 10.1038/ncomms8679 PMC4510653

[8] Huang J , Xiao D , Li G , et al. EphA2 promotes epithelial‐mesenchymal transition through the Wnt/beta‐catenin pathway in gastric cancer cells. Oncogene. 2014;33:2737‐2747. 23752181 10.1038/onc.2013.238

[9] Chen X , Wang X , Ruan A , et al. miR‐141 is a key regulator of renal cell carcinoma proliferation and metastasis by controlling EphA2 expression. Clin Cancer Res. 2014;20:2617‐2630. 24647573 10.1158/1078-0432.CCR-13-3224

[10] Naudin C , Sirvent A , Leroy C , et al. SLAP displays tumour suppressor functions in colorectal cancer via destabilization of the SRC substrate EPHA2. Nat Commun. 2014;5:3159. 24457997 10.1038/ncomms4159

[11] Huang C , Yuan W , Lai C , et al. EphA2‐to‐YAP pathway drives gastric cancer growth and therapy resistance. Int J Cancer. 2019;146(2020):1937–1949. 31376289 10.1002/ijc.32609

[12] Tadesse S , Yu M , Kumarasiri M , Le BT , Wang S . Targeting CDK6 in cancer: state of the art and new insights. Cell cycle (Georgetown, Tex). 2015;14:3220‐3230. 10.1080/15384101.2015.1084445 PMC4825601 26315616

[13] Uras IZ , Walter GJ , Scheicher R , et al. Palbociclib treatment of FLT3‐ITD+ AML cells uncovers a kinase‐dependent transcriptional regulation of FLT3 and PIM1 by CDK6. Blood. 2016;127:2890‐2902. 27099147 10.1182/blood-2015-11-683581 PMC4920675

[14] Andisheh‐Tadbir A , Ashraf MJ , Jeiroodi N . Expression of CDK6 in oral squamous cell carcinomas. Asian Pac J Cancer Prev. 2018;19:1013‐1016. 29693970 10.22034/APJCP.2018.19.4.1013 PMC6031802

[15] Shah M , Nunes MR , Stearns V . CDK4/6 inhibitors: game changers in the management of hormone receptor‐positive advanced breast cancer? Oncology (Williston Park, NY). 2018;32:216‐222. PMC6424488 29847850

[16] Goel S , DeCristo MJ , McAllister SS , Zhao JJ . CDK4/6 inhibition in cancer: beyond cell cycle arrest. Trends Cell Biol. 2018;28:911‐925. 30061045 10.1016/j.tcb.2018.07.002 PMC6689321

[17] Sheng Y , Wei J , Zhang Y , et al. Mutated EPHA2 is a target for combating lymphatic metastasis in intrahepatic cholangiocarcinoma. Int J Cancer. 2019;144:2440‐2452. 30412282 10.1002/ijc.31979

[18] Sun W‐L , Chen J , Wang Y‐P , Zheng H . Autophagy protects breast cancer cells from epirubicin‐induced apoptosis and facilitates epirubicin‐resistance development. Autophagy. 2011;7:1035‐1044. 21646864 10.4161/auto.7.9.16521

[19] Wu D , Suo Z , Kristensen GB , et al. Prognostic value of EphA2 and EphrinA‐1 in squamous cell cervical carcinoma. Gynecol Oncol. 2004;94:312‐319. 15297167 10.1016/j.ygyno.2004.05.019

[20] Holm R , de Putte GV , Suo Z , Lie AK , Kristensen GB . Expressions of EphA2 and EphrinA‐1 in early squamous cell cervical carcinomas and their relation to prognosis. Int J Med Sci. 2008;5:121‐126. 18566674 10.7150/ijms.5.121 PMC2424178

[21] Lamouille S , Xu J , Derynck R . Molecular mechanisms of epithelial‐mesenchymal transition. Nat Rev Mol Cell Biol. 2014;15:178‐196. 24556840 10.1038/nrm3758 PMC4240281

[22] Qureshi R , Arora H , Rizvi MA . EMT in cervical cancer: its role in tumour progression and response to therapy. Cancer Lett. 2015;356:321‐331. 25281477 10.1016/j.canlet.2014.09.021

[23] Wang H , Nicolay BN , Chick JM , et al. The metabolic function of cyclin D3‐CDK6 kinase in cancer cell survival. Nature. 2017;546:426‐430. 28607489 10.1038/nature22797 PMC5516959

[24] Sherr CJ , Beach D , Shapiro GI . Targeting CDK4 and CDK6: from discovery to therapy. Cancer Discov. 2016;6:353‐367. 26658964 10.1158/2159-8290.CD-15-0894 PMC4821753

[25] Murphy CG , Dickler MN . The role of CDK4/6 inhibition in breast cancer. Oncologist. 2015;20:483‐490. 25876993 10.1634/theoncologist.2014-0443 PMC4425391

[26] Yu J , Zhao Y , Liu C , et al. Synergistic anti‐tumor effect of paclitaxel and miR‐34a combined with ultrasound microbubbles on cervical cancer in vivo and in vitro. Clin Transl Oncol. 2020;22:60‐69. 31093891 10.1007/s12094-019-02131-w

[27] Xiong Y , Li T , Assani G , et al. Ribociclib, a selective cyclin D kinase 4/6 inhibitor, inhibits proliferation and induces apoptosis of human cervical cancer in vitro and in vivo. Biomed Pharmacother. 2019;112:108602. 30784916 10.1016/j.biopha.2019.108602

[28] Ray A , Jena S , Dash B , et al. Hedychium coronarium extract arrests cell cycle progression, induces apoptosis, and impairs migration and invasion in HeLa cervical cancer cells. Cancer Manag Res. 2019;11:483‐500. 30655700 10.2147/CMAR.S190004 PMC6322495

