# Supplementary material for: EphA2 promotes tumorigenicity of cervical cancer by up‐regulating CDK6
Source: J Cell Mol Med. 2021 Feb 14;25(6):2967–75. doi: 10.1111/jcmm.16337 (PMC7957165; doi:10.1111/jcmm.16337)
Supplement: Supplementary file 1 — Supplementary Material [file JCMM-25-2967-s001.docx]

**Supplementary Information**

**EphA2 promotes tumorigenicity of cervical cancer by upregulating CDK6**

Changhao Huang, Zihua Chen, Zhengxi He, Zhenying Ban, Yuanhang Zhu, Leilei Ding, Chen Yang, Ji-hak Jeong, Weijie Yuan, and Li Yang


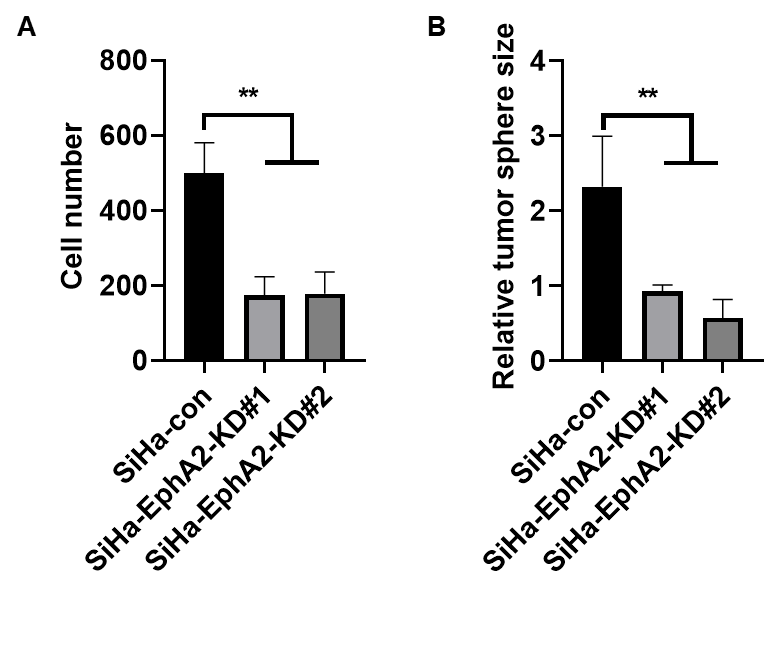


**Figure S1, Related to Figure 1. Quantification of cell number and relative tumor sphere size.**

1. Quantification of cell number from Figure 1C. Cell number was quantified from transwell migration assay in SiHa cells transfected with EphA2 expression plasmid or control vector.
2. Quantification of relative tumor sphere size from Figure 1E. Relative tumor sphere size was quantified from tumor sphere formation assay in SiHa cells transfected with EphA2 shRNA or control shRNA.

The results in (A) and (B) represent the mean ± SD. Statistically significant differences are indicated. ***p* < 0.01.


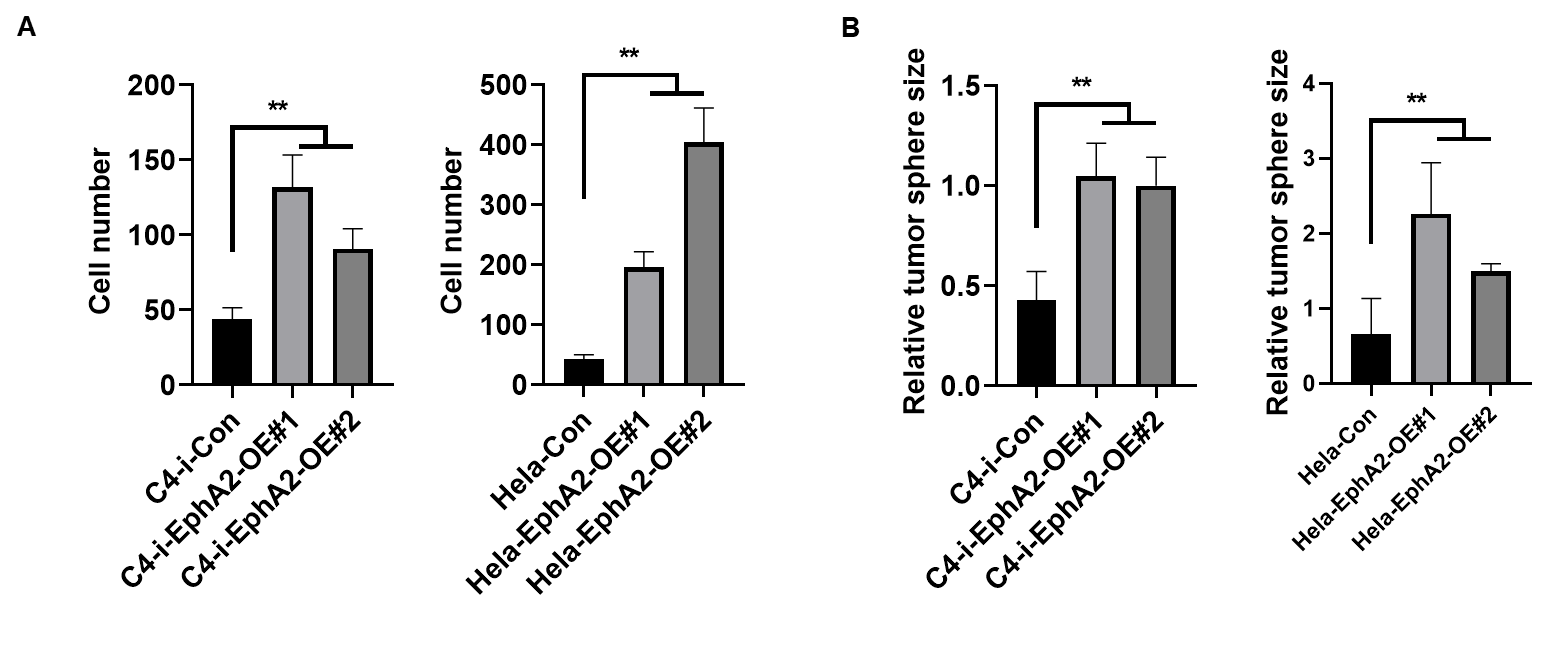


**Figure S2, Related to Figure 2. Quantification of cell number and relative tumor sphere size.**

1. Quantification of cell number from Figure 2C. Cell number was quantified from transwell migration assay in C4-i (left) or Hela cells (right) transfected with EphA2 expression plasmid or control vector.
2. Quantification of relative tumor sphere size from Figure 2E. Relative tumor sphere size was quantified from tumor sphere formation assay in C4-i (left) or Hela cells (right) transfected with EphA2 expression plasmid or control vector.

The results in (A) and (B) represent the mean ± SD. Statistically significant differences are indicated. ***p* < 0.01.

**
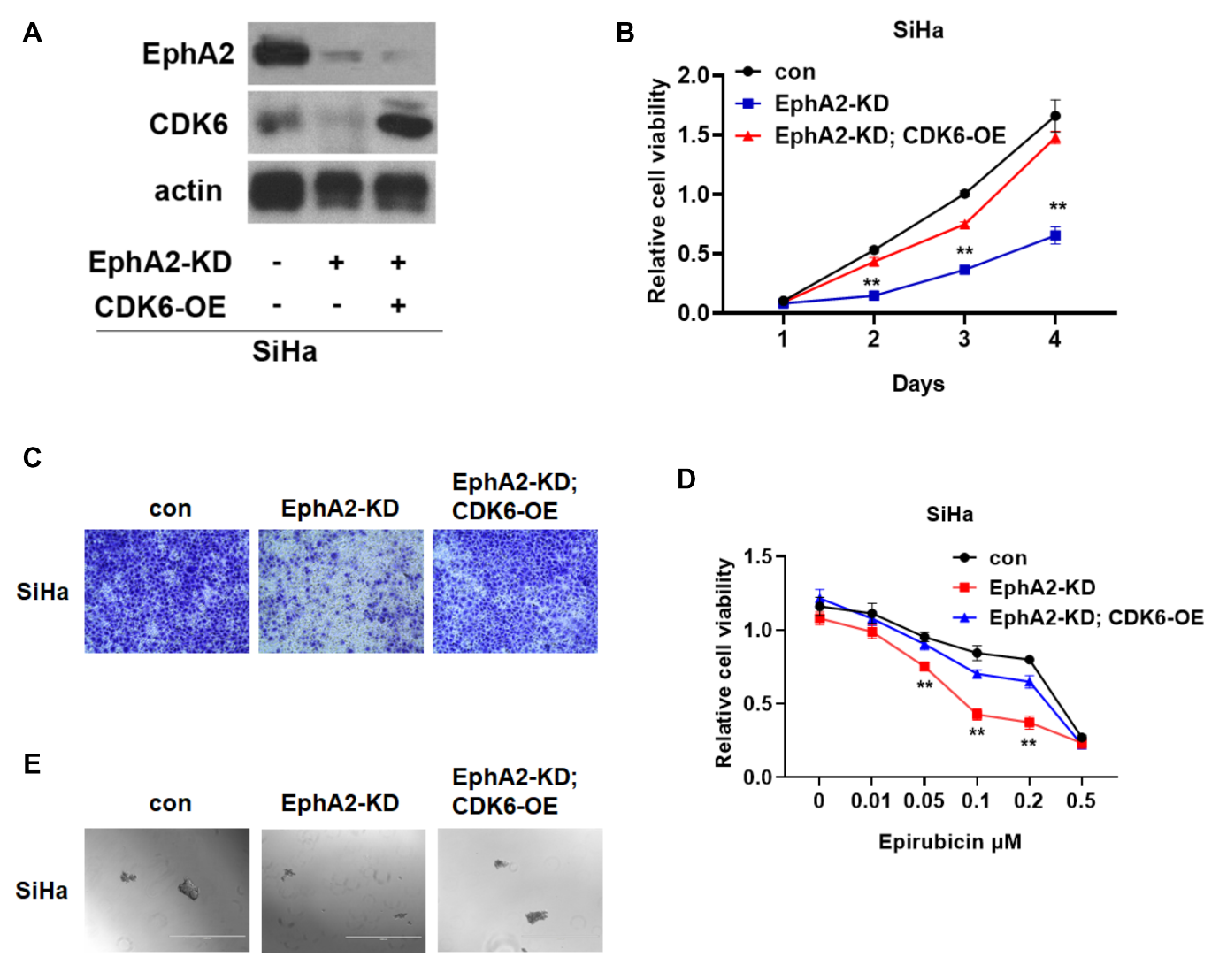
**

**Figure S3. CDK6 overexpression in EphA2 knockdown cells restores oncogenic activity of the cervical cancer cells.**

1. Western blot analysis of EphA2 and CDK6 protein expression in control, EphA2-KD, and EphA2-KD/CDK6-OE cells.
2. MTT analysis for cell proliferation of control, EphA2-KD, and EphA2-KD/CDK6-OE cells.
3. Transwell migration assay of control, EphA2-KD, and EphA2-KD/CDK6-OE cells.
4. Tumor sphere assay of control, EphA2-KD, and EphA2-KD/CDK6-OE cells. The scale bars represent 1000 μm.
5. MTT analysis for cell proliferation of the control, EphA2-KD, and EphA2-KD/CDK6-OE cells treated with various concentration of epirubicin.

The results in (B) and (E) represent the mean ± SD. Statistically significant differences are indicated. ***p* < 0.01.
